# Supplementary material for: The cortical representation of language timescales is shared between reading and listening
Source: Commun Biol. 2024 Mar 7;7:284. doi: 10.1038/s42003-024-05909-z (PMC11245628; doi:10.1038/s42003-024-05909-z)
Supplement: Supplementary file 2 — Supplemental Materials [file 42003_2024_5909_MOESM2_ESM.pdf]

Supplementary Information

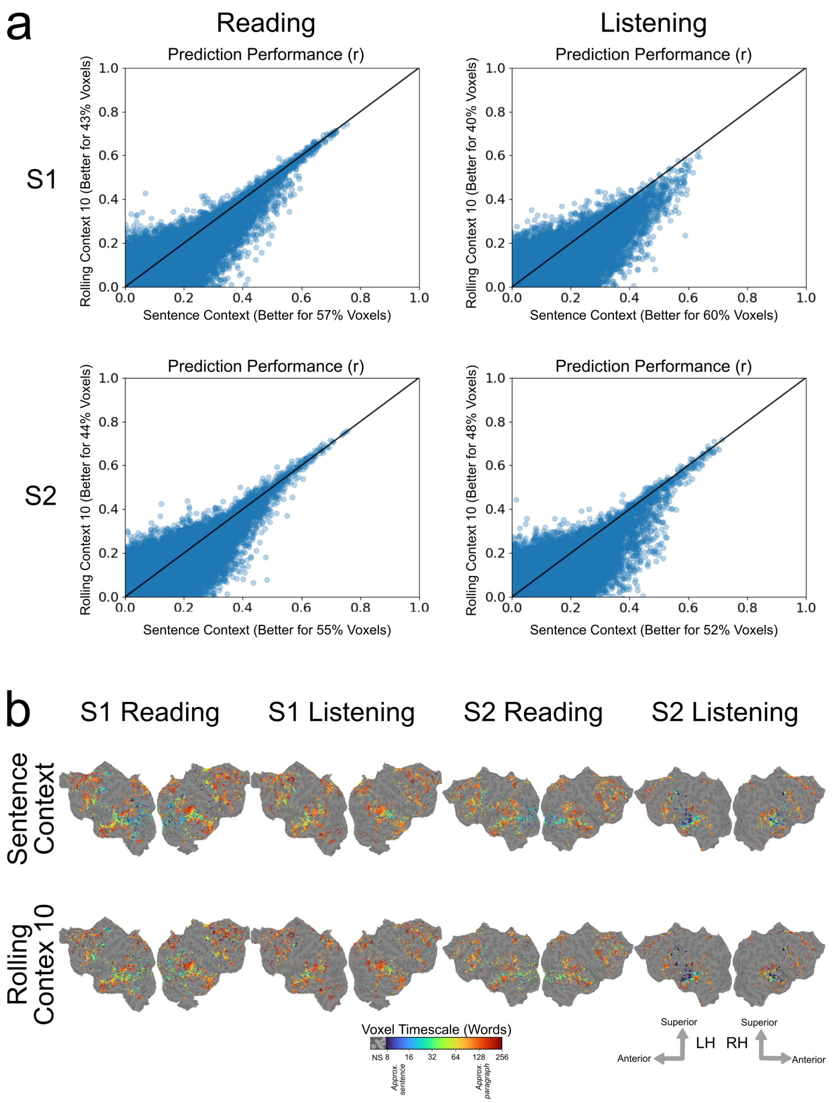

Figure S1: **Comparison of embedding extraction methods, sentence input context vs fixed rolling input context.** a. Encoding model prediction performance (r) obtained from a sentence-split input context (x-axis), and from a rolling input context of 10 words (y-axis). Each point represents one voxel. Axis labels indicate the percentage of voxels for which the respective embedding extraction method produces better performance (some voxels are predicted similarly well with both embedding extraction methods; therefore percentages may not sum to 100). A sentence-split input context produces more accurate predictions of brain responses than a rolling input context of 10 words. b. Timescale selectivity is shown for two representative participants (S1 and S2) and for reading and listening separately. Timescale selectivity is shown according to the color scale at the bottom. Voxels that were not significantly predicted are shown in grey (one-sided permutation test, $p < .05$, FDR corrected). For both embedding extraction methods, temporal cortex contains a spatial gradient from intermediate to long timescale selectivity along the superior to inferior axis, prefrontal cortex (PFC) contains a spatial gradient from intermediate to long timescale selectivity along the posterior to anterior axis, and precuneus is predominantly selective for long timescales. While Panel A shows that a sentence-split input context length produces more accurate models of brain responses, estimates of timescale selectivity are robust to the input context method.

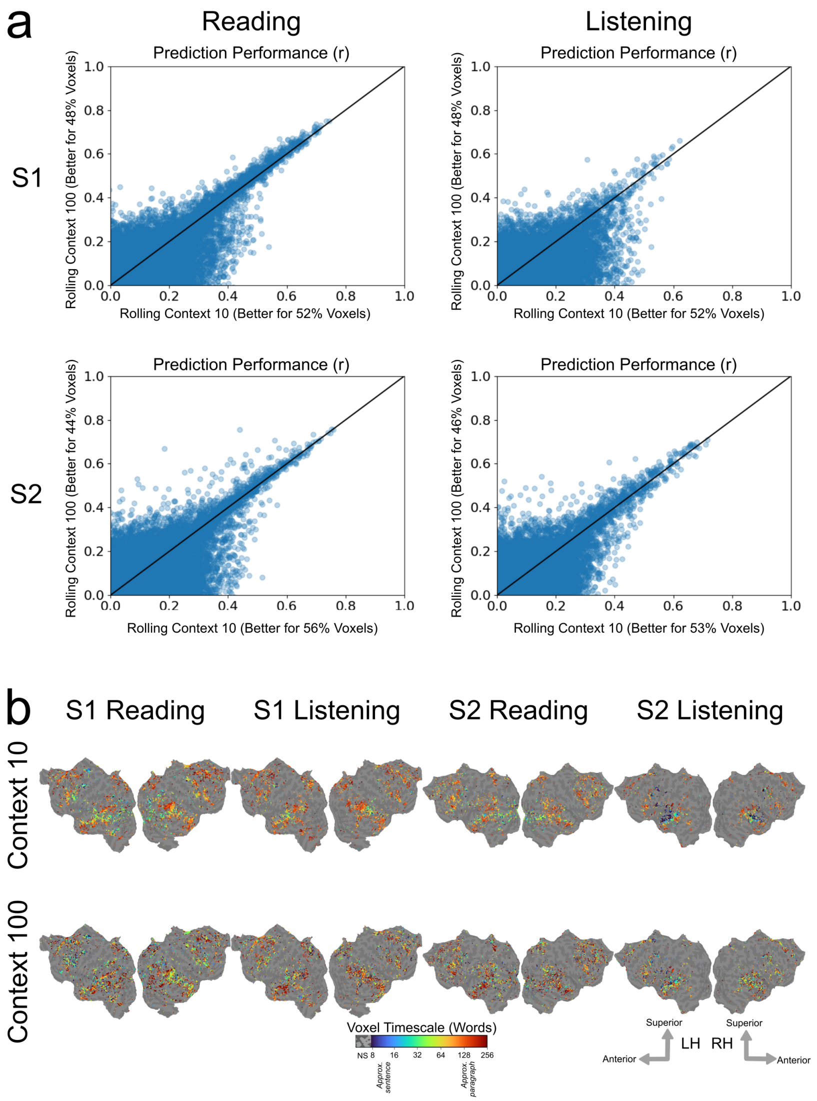

Figure S2: **Comparison of embedding extraction methods, input context length 10 words vs input context length 100 words.** a. Encoding model prediction performance (r) using a rolling input context of 10 words (x-axis) vs a rolling input context of 100 words (y-axis). Each point represents one voxel. Axis labels indicate the number of voxels for which the respective embedding extraction method produces better performance (some voxels are predicted similarly well with both embedding extraction methods; therefore, percentages may not sum to 100.) An input context of 10 words produces more accurate predictions of brain responses than an input context of 100 words. b. Timescale selectivity is shown for two representative participants (S1 and S2), for reading and listening separately. For each significantly predicted voxel, timescale selectivity is shown according to the color scale at the bottom. Voxels that were not significantly predicted are shown in grey (one-sided permutation test, $p < .05$, FDR corrected). For both embedding extraction methods, temporal cortex contains a spatial gradient from intermediate to long timescale selectivity along the superior to inferior axis, prefrontal cortex (PFC) contains a spatial gradient from intermediate to long timescale selectivity along the posterior to anterior axis, and precuneus is predominantly selective for long timescales. These results suggest using a shorter input context produces more accurate models of brain responses, and that estimates of timescale selectivity are similar between different input context lengths.

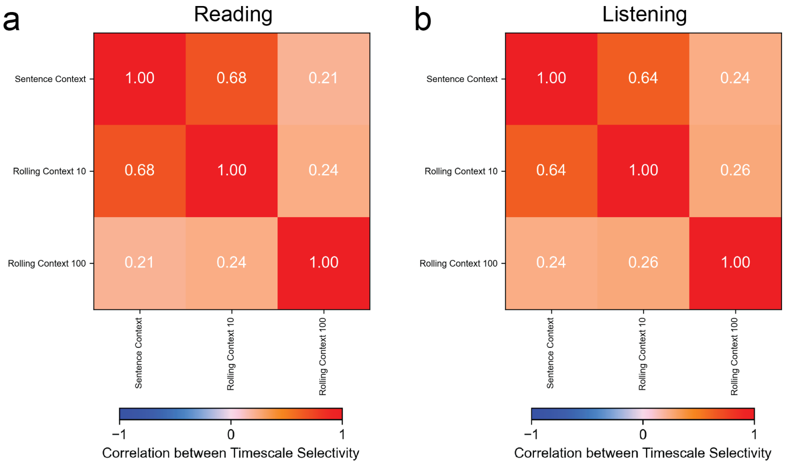

Figure S3: **Comparison of estimated timescale selectivity between different input context lengths.** Timescale selectivity was estimated separately with a sentence-length stimulus input context, a rolling 10-word input context, and a rolling 100-word input context. For each pair of input contexts, group-averaged voxelwise spatial correlation between estimated timescale selectivity is shown, for reading (a) and listening (b) separately. The sentence-length context and rolling 10-word context produce correlated estimates of timescale selectivity. The rolling 100-word context produces estimates of timescale selectivity that are less similar, but still positively correlated with estimates from the other two embedding methods. Furthermore, the similarity between sentence-length and rolling input contexts suggests that the inclusion of future context in sentence-length input contexts does not qualitatively change estimates of timescale selectivity.

	2-4 words	4-8 words	8-16 words	16-32 words	32-64 words	64-128 words	128-256 words	256+ words
S1	0.003*	<0.001*	0.004*	<0.001*	0.062	1	0.012*	0.001*
S2	<0.001*	<0.001*	<0.001*	<0.001*	0.048*	0.006*	0.038*	0.008*
S3	<0.001*	<0.001*	0.071	0.004*	0.039*	<0.001*	<0.001*	0.002*
S4	<0.001*	0.001*	0.008*	<0.001*	0.062	0.246	0.021*	0.005*
S5	<0.001*	<0.001*	0.563	0.001*	0.48	0.21	<0.001*	0.425
S6	<0.001*	<0.001*	0.001*	0.01*	0.658	0.043*	0.003*	0.043*
S7	<0.001*	<0.001*	<0.001*	0.092	0.088	0.001*	0.08	0.007*
S8	<0.001*	<0.001*	0.062	0.091	0.018*	0.157	0.003*	0.07
S9	<0.001*	<0.001*	0.001*	<0.001*	0.036*	<0.001*	<0.001*	<0.001*

Table S1: **Significance of correlation between selectivity for each timescale during reading and listening.** For each timescale and participant, the Pearson correlation coefficient was computed across voxels between selectivity for that timescale during reading and listening (Figure 4). The p-value of each correlation is shown here. Asterisks indicate p-values that are significant (one-sided permutation test, $p < .05$, FDR corrected with a Benjamini-Hochberg correction for multiple comparisons).

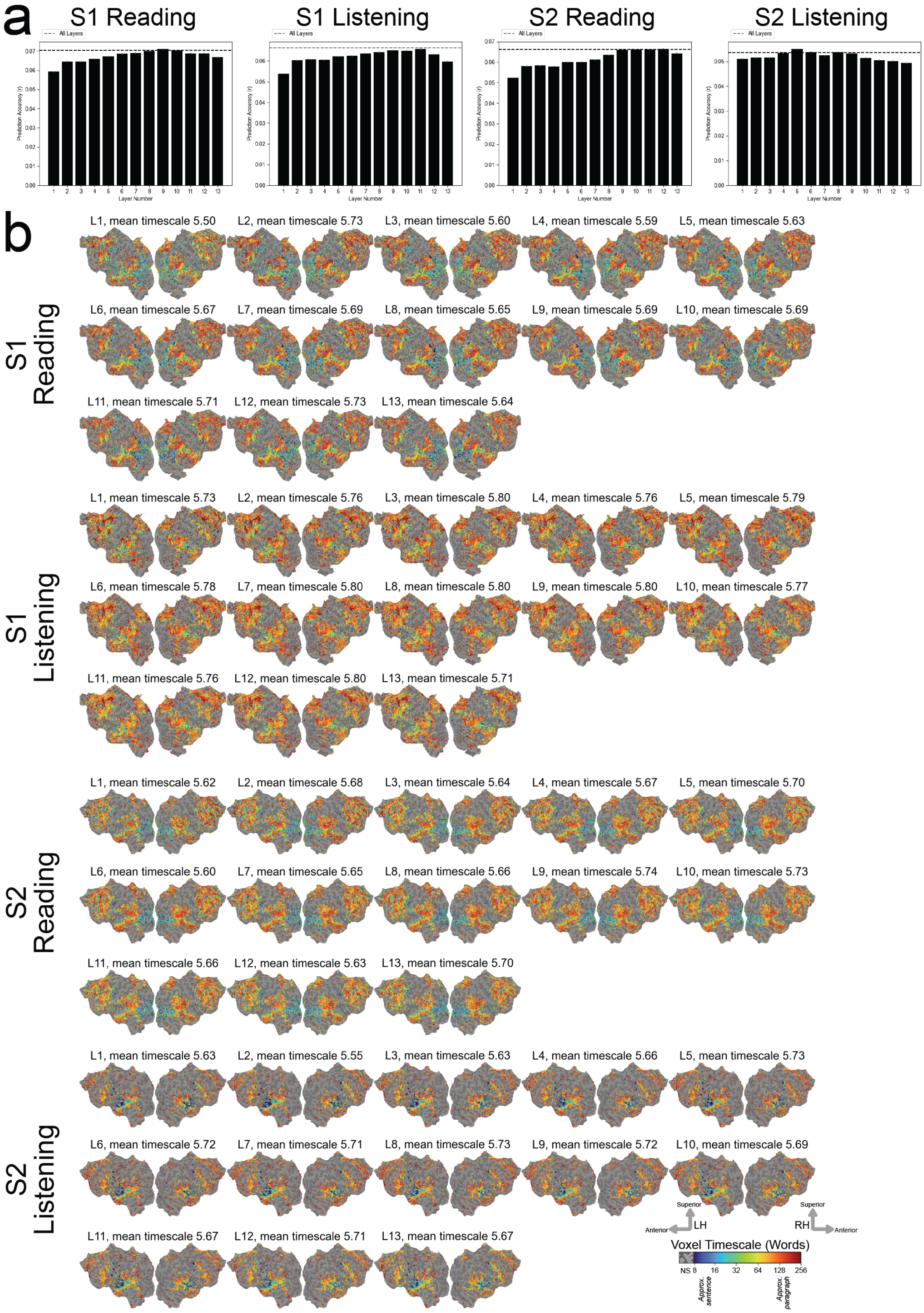

Figure S4: **Comparison of embedding extraction methods, all layers of BERT vs only a single layer.** Timescale selectivity was estimated with embeddings from each layer of BERT. Results are shown for two representative participants (S1 and S2). a. Bars denote number of significantly predicted voxels for each layer. Errorbars denote standard error. The dashed line denotes the number of significantly predicted voxels obtained from using all layers of BERT together. Including all layers of BERT generally produces better predictions of brain responses than only using a single layer. b. Timescale selectivity estimated with embeddings from each layer separately. For each significantly predicted voxel, timescale selectivity is shown according to the color scale at the bottom. Voxels that were not significantly predicted are shown in grey (one-sided permutation test, $p < .05$, FDR corrected). Estimates of timescale selectivity are similar between the different embedding methods – spatial gradients from intermediate to long timescale selectivity are found along the superior to inferior axis of temporal cortex and along the posterior to anterior axis of prefrontal cortex (PFC), and precuneus is predominantly selective for long timescales. Embeddings from only a single layer of BERT often result in slightly worse prediction performance, but the choice of layer does not substantially affect estimates of timescale selectivity.

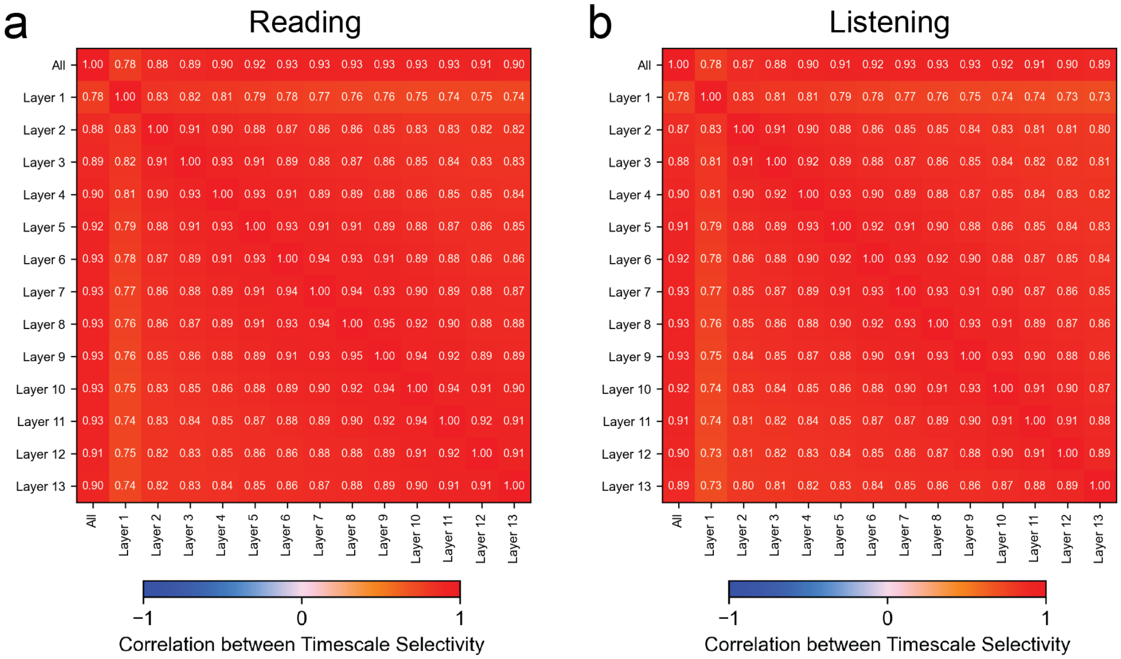

Figure S5: **Comparison of estimated timescale selectivity between different embedding layers.** Timescale selectivity was estimated separately with embeddings from each layer of BERT. Group-averaged voxelwise spatial correlation between timescale selectivity is shown for each pair of layers, for reading (a) and listening (b) separately. Estimates of timescale selectivity are highly correlated across layers. Estimates are more similar for layers that are closer together, suggesting a small effect of stimulus layer on estimates of timescale selectivity. Overall, estimates of timescale selectivity are consistent across different layers.

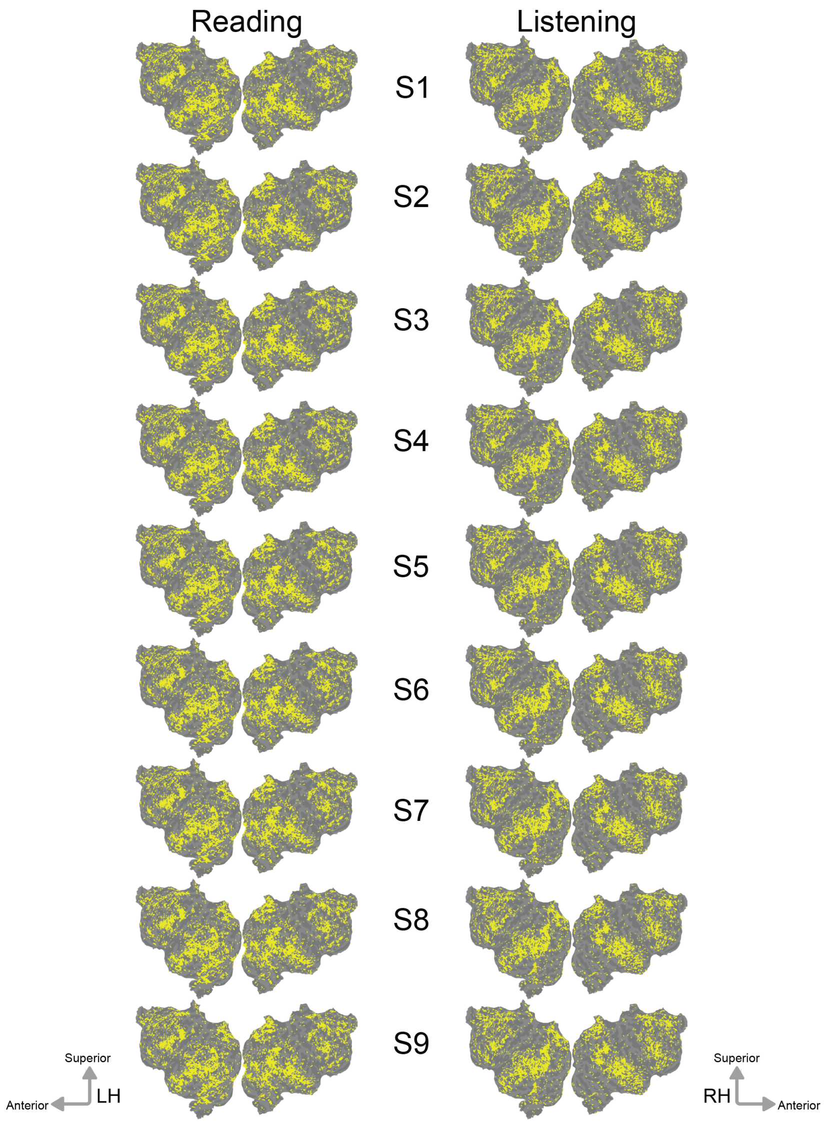

Figure S6: **Language-selective voxels in each modality.** The set of language-selective voxels is shown for reading and listening separately on the flattened cortical surface of each participant. Language-selective voxels are shown in yellow. For both modalities, voxels across temporal, parietal, and prefrontal cortices are language-selective.

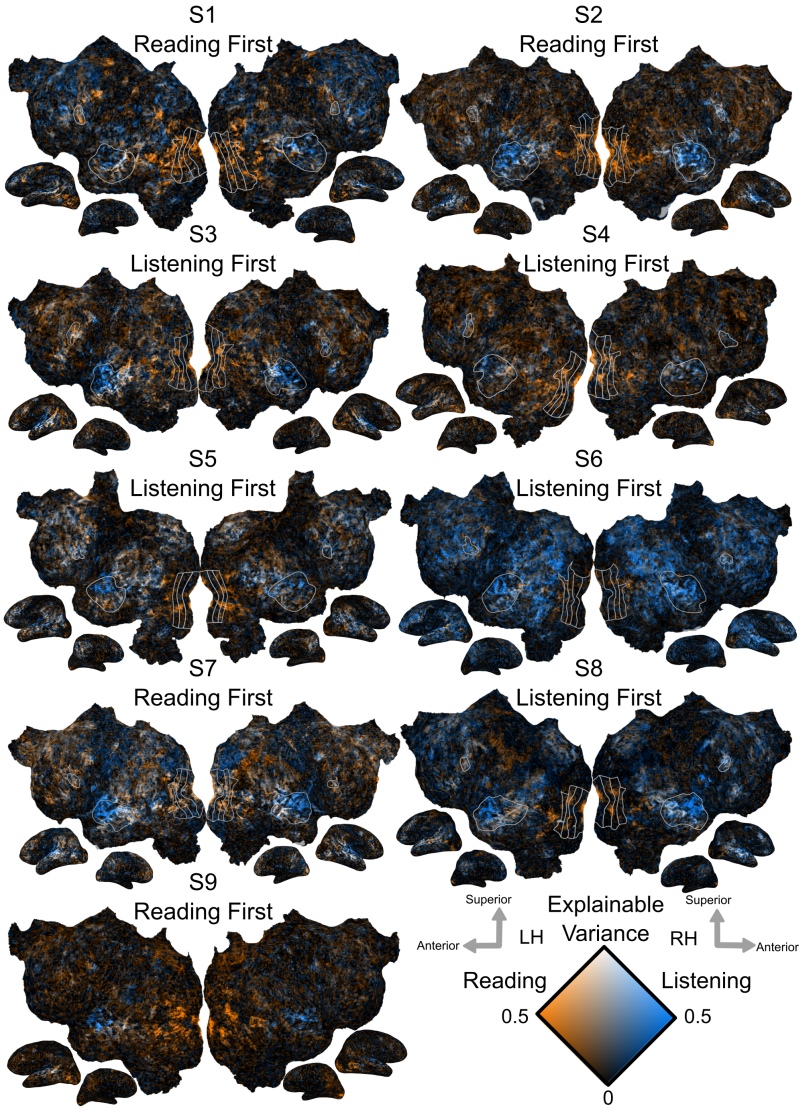

Figure S7: **Explainable variance in reading vs listening.** The explainable variance (EV) for reading and listening is shown on the flattened cortical surface of each participant. The first presented modality for each participant is indicated by the figure subtitles. Orange voxels had high EV for reading. Blue voxels had high EV for listening. White voxels had high EV for both modalities. The stimulus modality with stronger EV varies across participants, possibly due to individual participant preferences for certain modalities or because of small differences in noise across sessions. The EV of a voxel is computed using the measured BOLD response in a voxel over N repetitions of a stimulus with T timepoints $y_1, ...y_N \in \mathbb{R}^T$ as follows (Y must be zscored across time):
$\bar{y} = \frac{1}{N}\sum_{i=1}^{N} y_i$
$r_i = y_i - \bar{y}$
$EV = \frac{1}{N}\sum_{i=1}^{N} Var(y_i) - \frac{N}{N-1}\sum_{i=1}^{N} Var(r_i)$

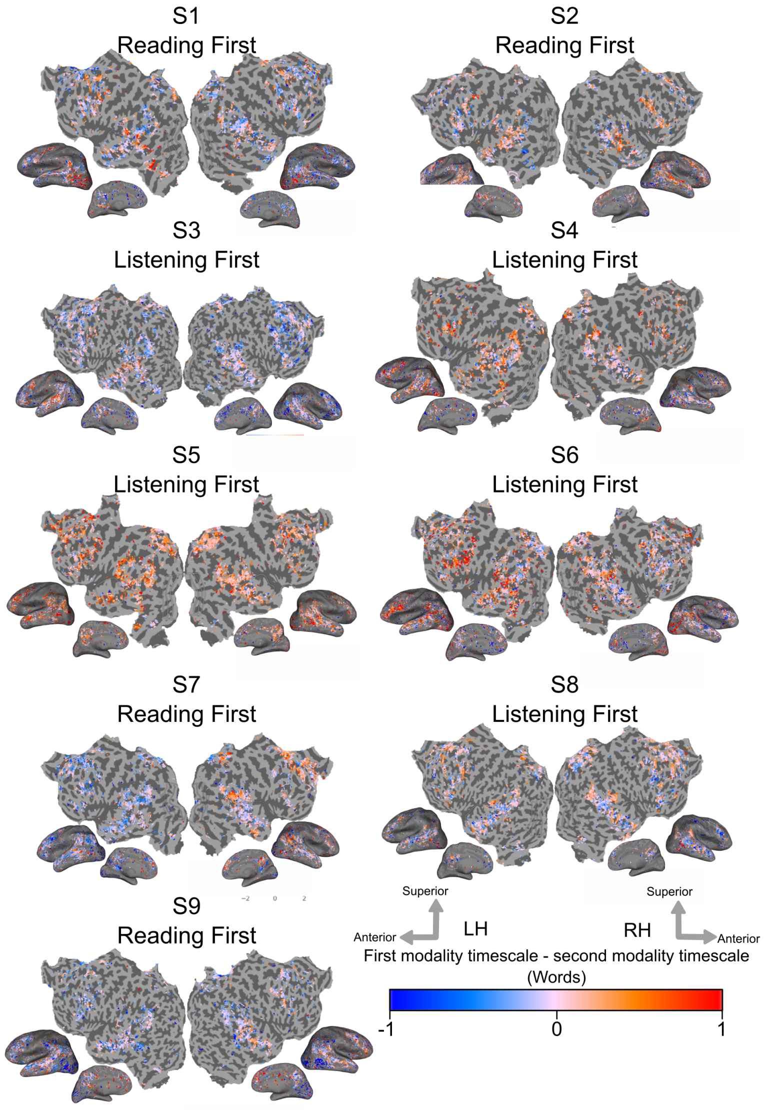

Figure S8: **Difference between timescale selectivity in first and second presented modalities.** The difference between timescale selectivity in the first and second presented modalities is shown on the flattened cortical surface of each participant. The first presented modality for each individual participant is indicated by the figure subtitles. Red voxels have longer timescale selectivity for the first presented modality. Blue voxels have longer timescale selectivity for the second presented modality. Voxels shown in grey were not significantly predicted in both modalities. Timescale selectivity is overall longer in the first presented modality than in the second presented modality in six participants (S1, S2, S4, S5, S6, S8; $p < .05$ by a two-sided t-test for paired samples). Voxel timescale selectivity is on average longer in the second presented modality than in the first presented modality in the other three participants (S3, S7, S9).

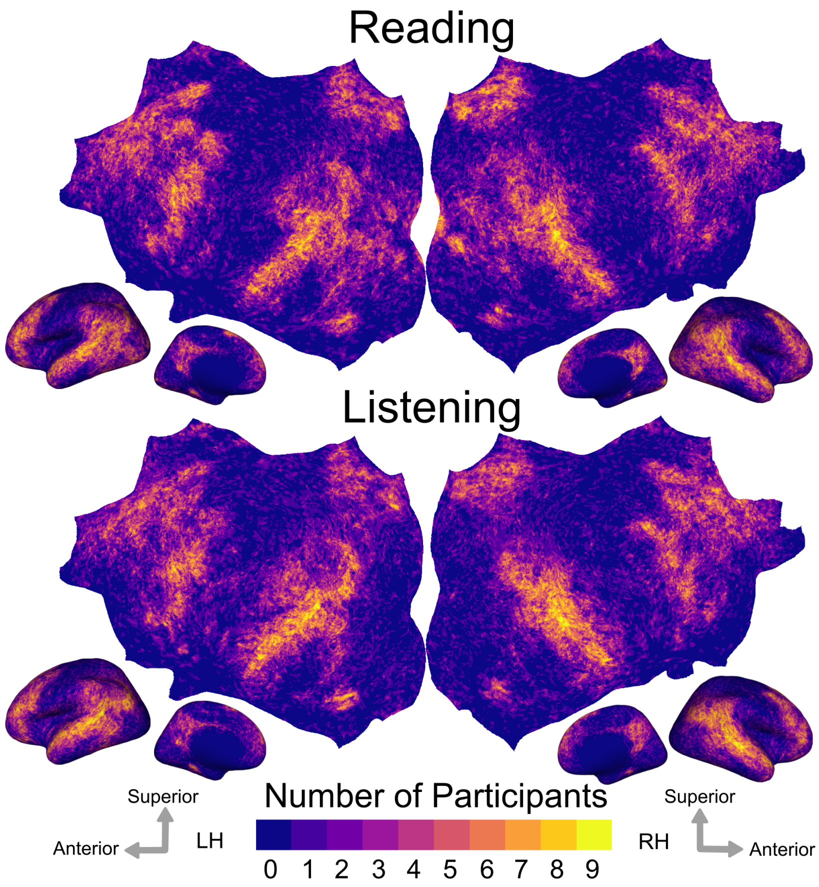

Figure S9: **Number of participants significantly predicted in each vertex.** The set of significantly predicted voxels in each participant was mapped to a standard fsAverage vertex space for reading and listening separately. The number of participants that was significantly predicted for each vertex is shown for reading (a) and listening (b) separately. The number of participants is indicated by the colorbar at the bottom. Brighter vertices are significantly predicted in more participants. Vertices across temporal, parietal, and prefrontal cortices are significantly predicted in most participants for both modalities.

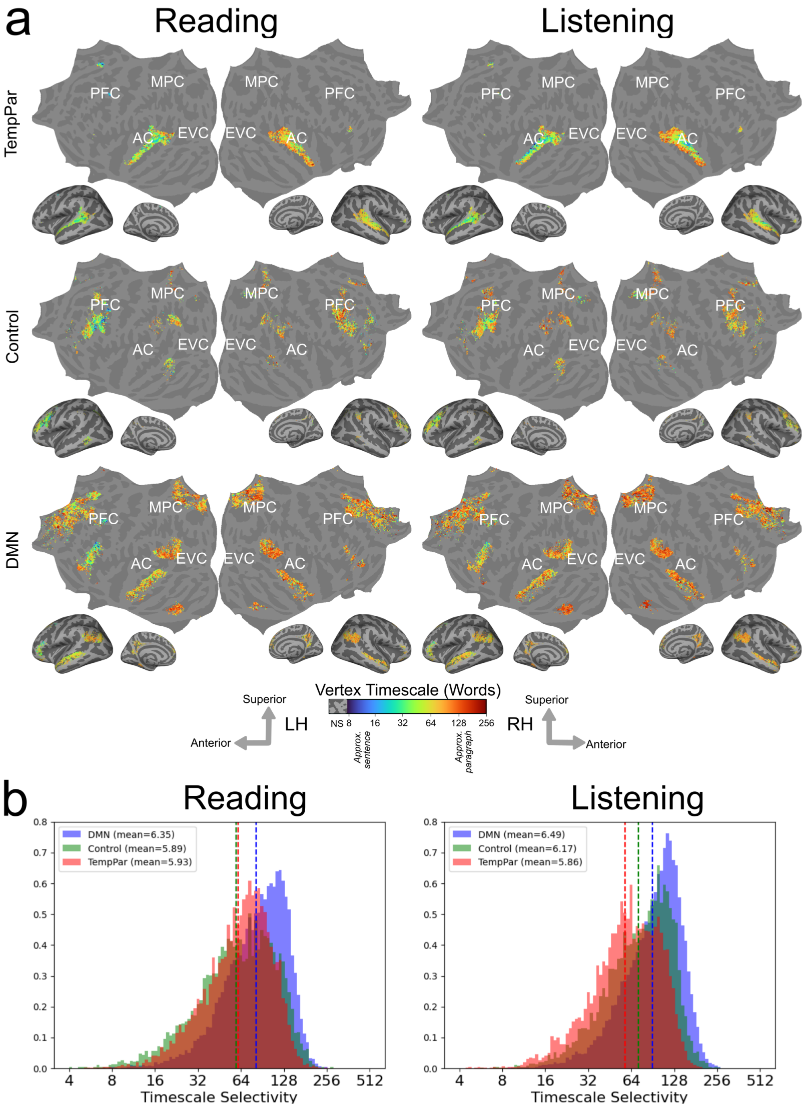

Figure S10: **Group-level timescale selectivity across previously proposed cortical networks.** The Yeo2011 cortical parcellation was used to determine 17 cortical parcels in fsAverage space[1]. From this parcellation pre-defined network labels were used to identify three proposed networks (temporo-parietal network (TempPar), cognitive control network (Control), and default mode network (DMN)). a. Group-level timescale selectivity is shown on the flattened cortical surface of the fsAverage template brain, for the three networks separately, and for reading and listening separately. Timescale selectivity is shown according to the color scale at the bottom. Voxels that were not significantly predicted for both modalities in at least three participants are shown in grey (one-sided permutation test, $p < .05$, FDR corrected). The temporo-parietal network (TempPar) contains a gradient from short to long timescale selectivity in superior to inferior temporal cortex. Prefrontal areas of the control network (Control) contain a mix of timescale selectivity. The default mode network (DMN) contains a gradient from short to long timescale selectivity from posterior to anterior prefrontal cortex. b. The distribution of group-level timescale selectivity for each network is shown for reading and listening separately. Histograms include vertices that were significantly predicted for both modalities in at least three participants. Each network is selective for a range of different timescales. The DMN contains longer timescale selectivity than the other networks. This difference was statistically significant for both reading and listening, at the group level and for eight of the nine individual participants ($p = 0.13$ for DMN vs TempPar S5 reading; $p < .01$ at the group level and for all other participants, modalities, and network pairs). The preference towards longer timescale selectivity within the default mode network is consistent with reports that long-timescale narrative-length information may be processed in this network[2].

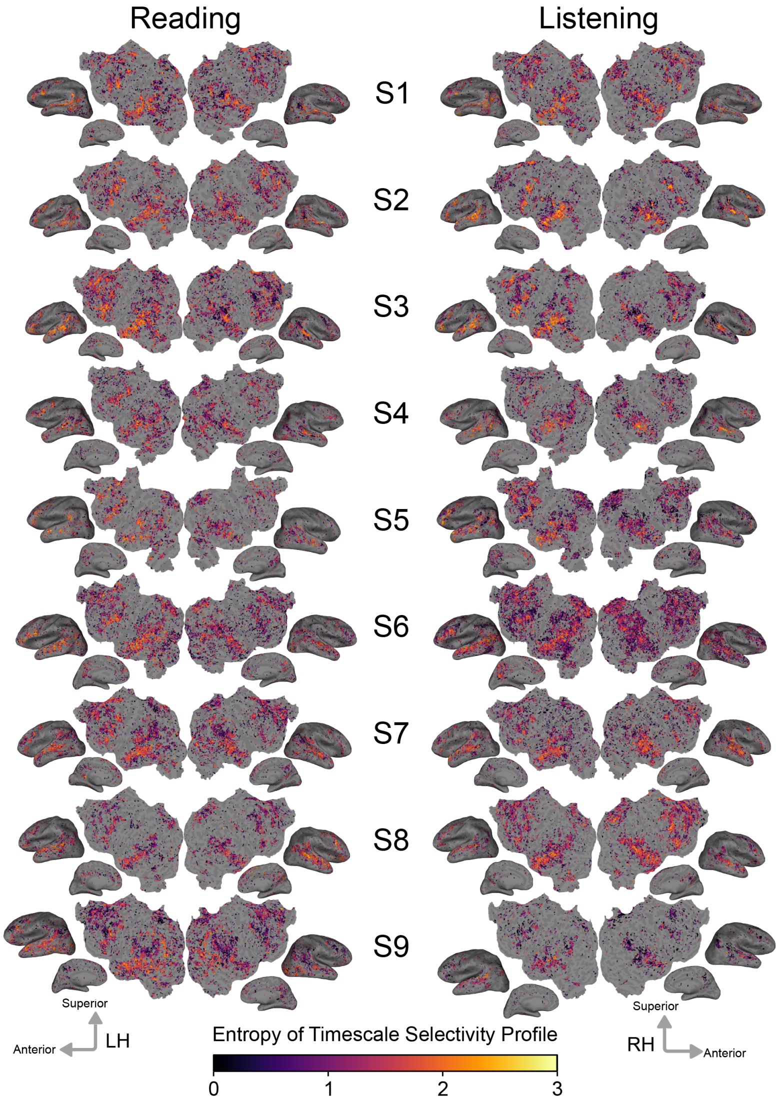

Figure S11: **Uniformity of timescale selectivity.** For each voxel, the uniformity of timescale selectivity was computed as the entropy of the timescale selectivity profile of the voxel. Voxels with more uniform timescale selectivity have a flatter timescale selectivity profile; thus, higher entropy corresponds to more uniform timescale selectivity, whereas lower entropy corresponds to more peaked timescale selectivity. The entropy of the timescale selectivity profile of each voxel is shown according to the color scale at the bottom on the flattened cortical surface of each participant, for reading and listening separately. Brighter voxels have timescale selectivity profiles with higher-entropy. Darker voxels have timescale selectivity profiles with lower-entropy. Voxels that were not significantly predicted are shown in grey (one-sided permutation test, $p < .05$, FDR corrected). Voxel timescale selectivity profiles have higher entropy (i.e., are more uniform) in superior temporal gyrus (STG) and posterior areas of prefrontal cortex (PFC), and have lower entropy (i.e., are more peaked) in lateral and medial parietal cortex. These results are consistent with[3], which found that areas near STS and posterior PFC displayed a wider range of temporal receptive windows than other brain areas, whereas areas such as precuneus displayed a smaller range of temporal receptive windows.

Supplementary References

1. Yeo, B. T. *et al.* The organization of the human cerebral cortex estimated by intrinsic functional connectivity. *Journal of neurophysiology* (2011).
2. Simony, E. *et al.* Dynamic reconfiguration of the default mode network during narrative comprehension. *Nature communications* **7,** 12141 (2016).
3. Lerner, Y., Honey, C. J., Silbert, L. J. & Hasson, U. Topographic mapping of a hierarchy of temporal receptive windows using a narrated story. *Journal of Neuroscience* **31,** 2906–2915 (2011).
