# Supplementary material for: The cortical representation of language timescales is shared between reading and listening
Source: Commun Biol. 2024 Mar 7;7:284. doi: 10.1038/s42003-024-05909-z (PMC11245628; doi:10.1038/s42003-024-05909-z)
Supplement: Supplementary file 3 — Reporting Summary [file 42003_2024_5909_MOESM3_ESM.pdf]

nature portfolio

Corresponding author(s): Fatma Deniz

Last updated by author(s): Jan 28, 2024

Reporting Summary

Nature Portfolio wishes to improve the reproducibility of the work that we publish. This form provides structure for consistency and transparency in reporting. For further information on Nature Portfolio policies, see our Editorial Policies and the Editorial Policy Checklist.

Statistics

For all statistical analyses, confirm that the following items are present in the figure legend, table legend, main text, or Methods section.

n/a	Confirmed	
☐	☒	The exact sample size (*n*) for each experimental group/condition, given as a discrete number and unit of measurement
☐	☒	A statement on whether measurements were taken from distinct samples or whether the same sample was measured repeatedly
☐	☒	The statistical test(s) used AND whether they are one- or two-sided<br>*Only common tests should be described solely by name; describe more complex techniques in the Methods section.*
☐	☒	A description of all covariates tested
☐	☒	A description of any assumptions or corrections, such as tests of normality and adjustment for multiple comparisons
☐	☒	A full description of the statistical parameters including central tendency (e.g. means) or other basic estimates (e.g. regression coefficient) AND variation (e.g. standard deviation) or associated estimates of uncertainty (e.g. confidence intervals)
☐	☒	For null hypothesis testing, the test statistic (e.g. *F*, *t*, *r*) with confidence intervals, effect sizes, degrees of freedom and *P* value noted<br>*Give P values as exact values whenever suitable.*
☒	☐	For Bayesian analysis, information on the choice of priors and Markov chain Monte Carlo settings
☒	☐	For hierarchical and complex designs, identification of the appropriate level for tests and full reporting of outcomes
☐	☒	Estimates of effect sizes (e.g. Cohen's *d*, Pearson's *r*), indicating how they were calculated

Our web collection on statistics for biologists contains articles on many of the points above.

Software and code

Policy information about availability of computer code

Data collection	This study used data that was previously collected for other studies (Huth et al., 2016, Deniz et al., 2019). The data were preprocessed with the FMRIB Linear Image Registration Tool (FLIRT) from FSL 5.0.
Data analysis	All model fitting and analysis was performed using custom software written in Python, making heavy use of NumPy (Harris et al., 2020), SciPy (Virtanen et al., 2020), Matplotlib (Hunter, 2007), Himalaya (Dupre la Tour et al., 2022), and Pycortex (Gao et al., 2015).

For manuscripts utilizing custom algorithms or software that are central to the research but not yet described in published literature, software must be made available to editors and reviewers. We strongly encourage code deposition in a community repository (e.g. GitHub). See the Nature Portfolio guidelines for submitting code & software for further information.

Data

Policy information about availability of data

All manuscripts must include a data availability statement. This statement should provide the following information, where applicable:

- Accession codes, unique identifiers, or web links for publicly available datasets
- A description of any restrictions on data availability
- For clinical datasets or third party data, please ensure that the statement adheres to our policy

This study made use of data originally collected for separate studies (Huth et al., 2016; de Heer et al., 2017; Deniz et al., 2019). The data can be accessed at https://gin.g-node.org/denizenslab/narratives_reading_listening_fMRI.

Human research participants

Policy information about studies involving human research participants and Sex and Gender in Research.

Reporting on sex and gender	Functional data was collected from six male participants and three female participants: S1 (male, age 31), S2 (male, age 31), S3 (female, age 28), S4 (female, age 25), S5 (male, age 30), S6 (male, age 25), and S7 (male, age 36), S8 (female, age 24), S9 (male, age 24).
Population characteristics	Functional data was collected on nine participants (six males and three females) between the ages of 24 and 36.
Recruitment	Study participants were graduate students and postdoctoral scholars at UC Berkeley who were trained to participate in long duration functional MRI experiments.<br>Two of the participants were authors on the Huth et al., 2016 and Deniz et al., 2019 papers (A.G.H. and A.O.N.-E.).
Ethics oversight	Committee for Protection of Human Subjects at the University of California, Berkeley provided oversight.

Note that full information on the approval of the study protocol must also be provided in the manuscript.

Field-specific reporting

Please select the one below that is the best fit for your research. If you are not sure, read the appropriate sections before making your selection.

☒ Life sciences ☐ Behavioural & social sciences ☐ Ecological, evolutionary & environmental sciences

For a reference copy of the document with all sections, see nature.com/documents/nr-reporting-summary-flat.pdf

Life sciences study design

All studies must disclose on these points even when the disclosure is negative.

Sample size	Data were collected on nine participants (six males and three females). Because the current study used a voxelwise encoding model framework, each participant's data were analyzed individually, and both statistical significance and out-of-set prediction accuracy (i.e., generalization) are reported for each participant separately. Because each participant provides a complete replication of all hypothesis tests, sample size calculations were neither required nor performed.
Data exclusions	No data was excluded.
Replication	We verified the reproducibility of the results by rerunning the analysis pipeline in each individual subject and in each dataset (listening and reading) separately.
Randomization	Listening and reading presentations were counterbalanced randomly across participants.
Blinding	There was not a treatment or control group. Both functional MRI experiments (listening and reading to stories) were done on each individual subject.

Reporting for specific materials, systems and methods

We require information from authors about some types of materials, experimental systems and methods used in many studies. Here, indicate whether each material, system or method listed is relevant to your study. If you are not sure if a list item applies to your research, read the appropriate section before selecting a response.

Materials & experimental systems

n/a	Involved in the study	
☒	☐	Antibodies
☒	☐	Eukaryotic cell lines
☒	☐	Palaeontology and archaeology
☒	☐	Animals and other organisms
☒	☐	Clinical data
☒	☐	Dual use research of concern

Methods

n/a	Involved in the study	
☒	☐	ChIP-seq
☒	☐	Flow cytometry
☐	☒	MRI-based neuroimaging

Magnetic resonance imaging

Experimental design

Design type	Task, naturalistic functional MRI experiment (Lisening to and RSVP reading of stories)
Design specifications	The stimuli consisted ten selected 10-15 min stories taken from The Moth Radio Hour and used previously (Huth et al., 2016). Each spoken and written story was presented during a separate fMRI scan. The length of each scan was the same as the story. Each subject These data were collected during 2-3 h scanning sessions that were performed on different days.<br>The held-out model validation dataset consisted of one 10 min story. This story was played twice for each participant (once during each scanning session), and then the two responses were averaged.
Behavioral performance measures	No behavioral measure was collected. Participants passively listened to or read natural stories.

Acquisition

Imaging type(s)	functional, structural
Field strength	3 Tesla
Sequence & imaging parameters	Functional scans were collected using gradient echo EPI water excitation pulse sequence with repetition time (TR) =2.0045 s, echo time (TE) = 31 ms, flip angle = 70 degrees, voxel size = 2.24 x 2.24 x 4.1 mm (slice thickness = 3.5 mm with 18% slice gap), matrix size = 100 x 100, and field of view = 224 x 224 mm. 30 axial slices were prescribed to cover the entire cortex and were scanned in interleaved order. A custom-modified bipolar water excitation radiofrequency (RF) pulse was used to avoid signal from fat.<br>Anatomical data were collected using a T1-weighted multi-echo MP-RAGE sequence on the same 3T scanner.
Area of acquisition	A whole brain scan was used.

Diffusion MRI ☐ Used ☒ Not used

Preprocessing

Preprocessing software	Each functional run was motion-corrected using the FMRIB Linear Image Registration Tool (FLIRT) from FSL 5.0 (Jenkinson and Smith, 2001; Jenkinson et al., 2002). The brain surface of each subject was reconstructed using Freesurfer (Fischl et al., 1999).
Normalization	To compute group-level estimates, the estimates for each individual participant were projected to the standard FreeSurfer fsaverage vertex space (Fischl et al., 1999).
Normalization template	FreeSurfer fsaverage vertex space
Noise and artifact removal	Low-frequency voxel response drift was identified using a third order Savitsky–Golay filter with a 120 s window. This drift was subtracted from the signal.
Volume censoring	n/a

Statistical modeling & inference

Model type and settings	Voxel-wise encoding models in individual participant and modality that predict brain recordings as a function of stimulus features.
Effect(s) tested	We test whether timescale-specific aspects of the stimuli are predictive of fMRI recordings.

Specify type of analysis: ☒ Whole brain ☐ ROI-based ☐ Both

Statistic type for inference (See Eklund et al. 2016)	voxel-wise
Correction	The Benjamini-Hochberg false discovery rate (FDR) procedure was used to correct the resulting p-values for multiple comparisons within each participant and modality.

Models & analysis

n/a	Involved in the study
☒	☐ Functional and/or effective connectivity
☒	☐ Graph analysis
☐	☒ Multivariate modeling or predictive analysis

Multivariate modeling and predictive analysis

Feature extraction: Stimulus words were passed sentence-by-sentence to a pre-trained BERT model to obtain an embedding of each word (Devlin et al., 2019). Linear filters were convolved with word embeddings to obtain features that reflect stimulus language information at different timescales. Full details are available in Section 4.4.1 ("Construction of timescale-specific feature spaces").

Model training: The extracted stimulus features were used to estimate a voxelwise encoding model that predicts BOLD responses in each voxel from the stimulus features. Following previous work (e.g., Huth et al., 2016; Deniz et al., 2019; Dupre la Tour et al., 2022; Toneva et al., 2022), ridge regularization was used to estimate the linear mapping in the voxelwise encoding model. Full details of model training are available in Section 4.4.4 ("Voxelwise encoding model fitting"), Section 4.4.5 ("Regularization hyperparameter selection"), and Section 4.4.6 ("Model estimation and evaluation").

Model evaluation: Each encoding model's predictions are evaluated by computing the Pearson's correlation coefficient between the held-out validation dataset and the model predictions. Full details of model evaluation are available in Section 4.4.6 ("Model estimation and evaluation").
